# Ramosetron Does Not Reduce the Analgesic Efficacy of Tramadol after Gynecological Laparoscopic Surgery

**DOI:** 10.1155/2019/9584748

**Published:** 2019-07-09

**Authors:** Yanghyun Kim, Sungwoo Kang

**Affiliations:** Department of Anesthesia and Pain Medicine, National Cancer Center, Goyang, Republic of Korea

## Abstract

**Background:**

The effect of ramosetron on the analgesic action of tramadol is not well known when ramosetron is added to intravenous-tramadol patient-controlled analgesia (PCA) and infused continuously. The aim of this randomized noninferiority study was to evaluate the effects of ramosetron on the analgesic action of tramadol when it is administered simultaneously in women undergoing laparoscopic gynecology who are receiving tramadol via IV PCA.

**Method:**

This study used a prospective, randomized, controlled, noninferiority clinical trial design and compared the analgesic effect of tramadol plus ramosetron with that of tramadol only. A total of 110 postoperative patients, who were using IV PCA tramadol, were randomly assigned either to a group receiving ramosetron (group R, n=49) or to a group that received the same volume of normal saline continuously (group N, n=51). Observation time points for cumulative tramadol consumption were the first hour, and every 4 h up to 12 h and then 24 h after surgery. Pain intensity at rest and during movement, coughing, and nausea scores, the analgesic and antiemetic doses used, side effects, and patient satisfaction were evaluated 1 and 24 h after surgery.

**Results:**

Groups R and N received, respectively, 88 ± 55 vs. 79 ± 42 mg tramadol (P=0.511) after 1 h, 211 ± 122 vs. 198 ± 109 mg cumulative tramadol (P=0.610) after 4 h, 244 ± 150 vs. 231 ± 134 mg cumulative tramadol (P= 0.793) after 8 h, 250 ± 156 vs. 247 ± 153 mg cumulative tramadol (P=0.972) after 12 h, and 294 ± 190 vs. 284 ± 178 mg cumulative tramadol (P=0.791) after 24 h, postsurgery. Tramadol plus ramosetron was shown to be not significantly inferior to tramadol alone in alleviating the postoperative pain.

**Conclusions:**

The analgesic effect of tramadol combined with ramosetron was found to be noninferior to tramadol alone for postoperative PCA after laparoscopic gynecologic surgery.

## 1. Introduction

Tramadol in patient-controlled analgesia (PCA) is known to provide effective analgesia for acute pain following major surgery [1, 2]. Tramadol has a dual mechanism of action, which acts as a weak opioid agonist and an inhibitor of reuptake of serotonin and norepinephrine [3]. Tramadol has several advantages over other opioids, including a low probability of cardiovascular or respiratory depression, urinary retention, addiction, and reduced gastrointestinal mobility [4]. However, tramadol has been reported to cause relatively more nausea and vomiting, which limits its use [5]. Due to this reason, the antiemetic drug, serotonin receptor antagonist, is often coadministered or mixed with tramadol in postoperative PCA for preventing postoperative nausea and vomiting (PONV). However, the combination of drugs can cause undesirable effects. Several studies have reported that ondansetron, a 5-HT_3_ serotonin receptor antagonist, can reduce the analgesic effect of tramadol due to drug interaction effects [6–8]. Randomized controlled studies evaluating the effect of ramosetron on the analgesic effect of tramadol are lacking. Kim et al. reported that a single, 0.3 mg ramosetron injection at anesthesia induction did not reduce the analgesic effect of tramadol [9]. However, in clinical settings, patients may receive multiple doses of ramosetron or infusion continuously. Therefore, the aim of this randomized noninferiority study was to evaluate the effects of ramosetron on the analgesic action of tramadol when it is administered simultaneously in women undergoing laparoscopic gynecology who are receiving tramadol via IV PCA.

The primary outcome in this study was the group difference in cumulative dose of tramadol 1, 4, 8, 12, and 24 h postoperatively. The secondary outcomes included pain score, incidence of nausea and vomiting, total rescue antiemetic drug use, patients' satisfaction, and frequency of side effects during the 24h postoperative period.

## 2. Materials and Methods

This study was approved by the Institutional Review Board of the National Cancer Center (IRB no. NCCCTS 11599) and was registered with the Korean Clinical Trials Registry (CRiS, http://cris.nih.go.kr, KCT 0001750). After obtaining written informed consent, we enrolled 110 female patients between 18 and 64 years of age with an American Society of Anesthesiologists (ASA) physical status of I or II, who were undergoing laparoscopic gynecologic surgery. Patients who were unable to use the intravenous PCA device unassisted, had used other antiemetics or steroids in the 24 h prior to surgery, were on antidepressants, had a known allergy to tramadol or ramosetron, or had epilepsy, alcoholism, obesity (body mass index > 35 kg/m^2^), severe renal or hepatic insufficiency, or severe cardiopulmonary disease were excluded. On the day before surgery, patients were familiarized with the IV PCA device (Hospira Gemstar Infusion Pump, Abbott Laboratories, USA) and the 100 mm visual analog scale (VAS) (0 mm = no pain, 100 mm = worst imaginable pain) for evaluating postoperative pain intensity.

This study used a randomized, placebo-controlled design. Patients were randomized into two groups according to the random selection of sealed envelopes: group R (tramadol and ramosetron) or group N (tramadol and saline). In group R, PCA pumps were filled with 900 mg tramadol (9 mg/mL) mixed with 0.6 mg ramosetron diluted to 100 mL with 0.9% saline. In group N, the PCA pump was filled with tramadol (9 mg/mL) in saline solution.

Endotracheal intubation was performed and intermittent positive ventilation was provided to maintain an EtCO2 of 35–40 mmHg. Anesthesia was maintained with continuous, target-controlled infusion of propofol and remifentanil infusion to maintain the bispectral index at 40–50. At the beginning of fascia closure, all patients were connected to a PCA device and received a loading dose of 27 mg tramadol. The study drugs were prepared by a nurse blinded to the study treatment. The PCA device was set with a demand dose of 9 mg (at 5 min intervals) and a daily maximal dose of 900 mg. No basal infusion was set. At the beginning of skin closure, propofol and remifentanil were stopped and the neuromuscular blockade was reversed using pyridostigmine with glycopyrrolate. The total amounts of remifentanil and propofol infused during anesthesia were recorded.

At the PACU, for any patients who did not experience satisfactory relief from pain following the above PCA regimen, a single 50 *μ*g IV dose of fentanyl was added and the total amounts of fentanyl were recorded. If the VAS score was > 4 after discharge from the recovery room, 30 mg Ketorolac tromethamine (Trolac®) was administered. For patients with severe nausea and vomiting, an IV dose of metoclopramide was injected and the total amounts of metoclopramide used were recorded. Pain intensity was evaluated on a 100 mm VAS (0 mm = no pain, 100 mm = worst imaginable pain). A single anesthesiologist evaluated pain intensity at rest, during movement, during coughing, nausea (using a 4-point scale: 0 = no nausea; 1 = mild nausea, no request for pharmacological rescue; 2 = moderate nausea, request for pharmacological rescue; and 3 = severe nausea-resistant pharmacological therapy) [10], vomiting (presence or absence), and 1 and 24 h postoperatively. Observation time points for tramadol consumption were the first hour, and every 4 h up to 12 h and then 24 h after surgery. An additional patient satisfaction questionnaire of the overall analgesic technique was completed 24 h after surgery (very satisfied, satisfied, adequate, unsatisfied, very satisfied).

The primary objective of this study was to determine whether an IV PCA with the ramosetron and tramadol combination was not inferior to tramadol alone for postoperative pain management. Based on a previous study [6], the cumulative 24 h consumption of tramadol was calculated as 1,388 (± 340) and 703 (± 266) mg in the ramosetron and normal saline groups, respectively. The 95% lower limit of the prespecified noninferiority margin (30%) was 158.2 mg. The calculated sample size was 49 patients per study group with a type I error rate of 0.05, with 55 patients per group, assuming a 10% dropout rate.

### 2.1. Statistical Analysis

Wilcoxon's rank sum tests for continuous variables were used for comparisons between ramosetron group and control group. And *χ*2 test and Fisher's exact test were used for categorical variables. The difference of cumulative tramadol consumption during the postoperative 24 hours between groups was used for noninferiority test. Statistical analysis was performed using SAS version 9.4 (SAS Institute, Inc., Cary, NC, USA). A *p* value of <0.05 was taken to indicate statistical significance.

## 3. Results

**Figure 1:**
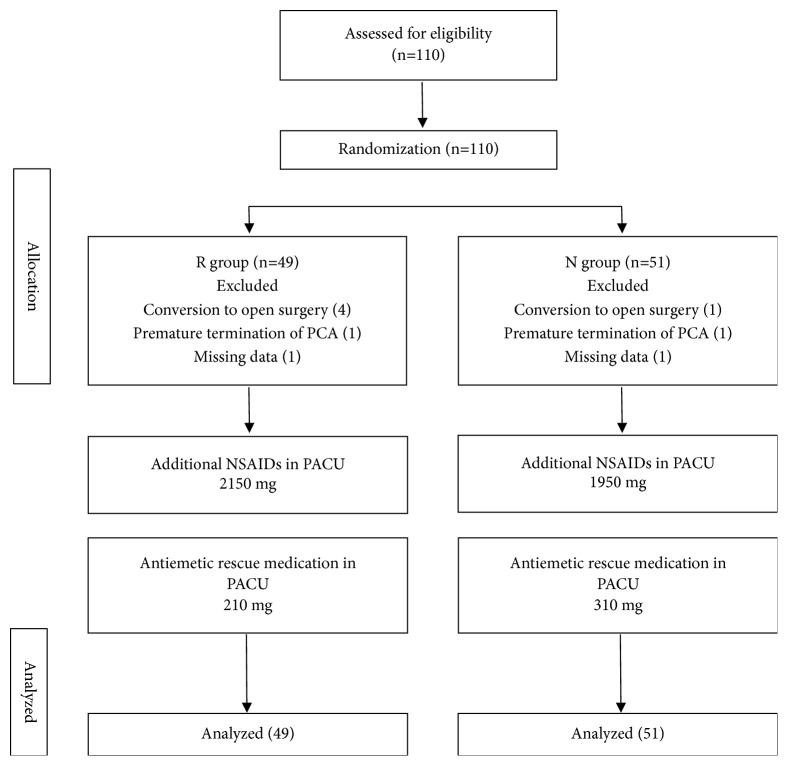
Study flow diagram.

This study recruited 110 patients in total, 10 of whom (4 in the normal saline group, 6 in the ramosetron group) were excluded because of missing data, conversion to open surgery, or stopping of the IV PCA due to persistent nausea (Figure 1). There were no significant group differences in the demographic data, ASA physical status, operation time, or the amount of remifentanil and propofol used (Table 1).

**Figure 2:**
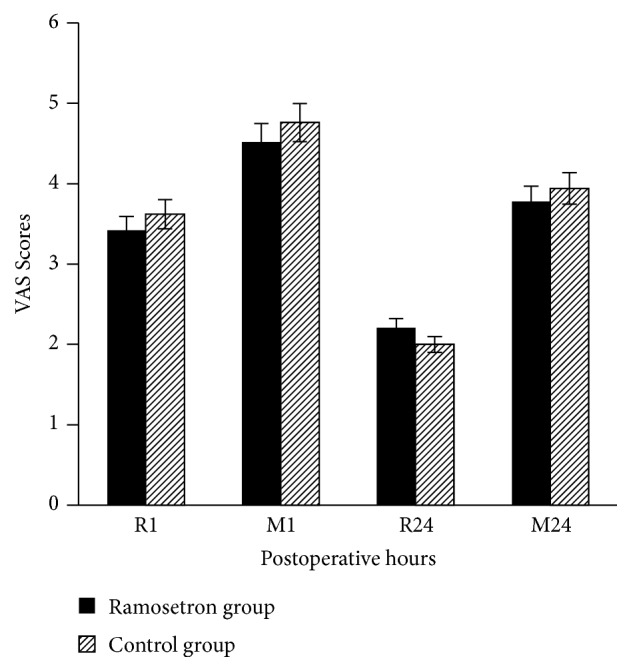
VAS scores in the postoperative 24 h. There were no differences between groups during the study period. R1: VAS score in the postoperative 1 h during movement, M1: VAS score in the postoperative 1 h during rest, R24: VAS score in the postoperative 24 h during rest, M24: VAS score in the postoperative 24 h during movement.

**Figure 3:**
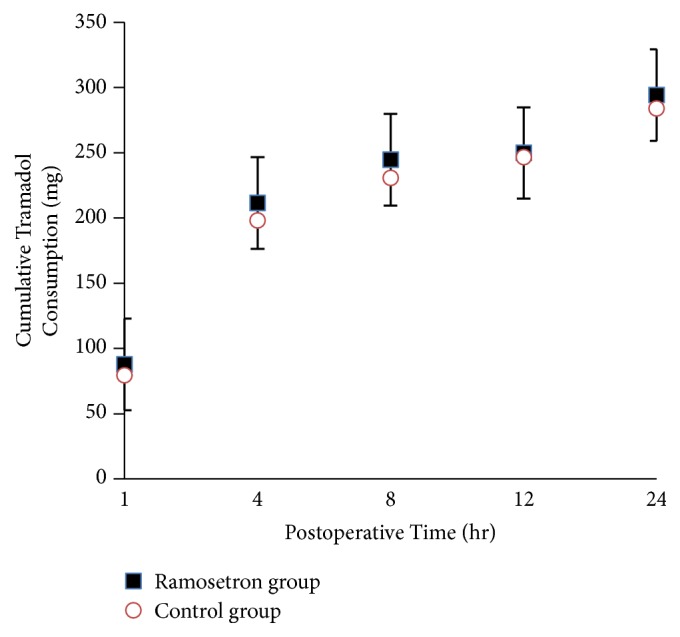
Cumulative tramadol consumption during the postoperative 24 hours.

Groups R and N received, respectively, 88 ± 55 vs. 79 ± 42 mg tramadol (P=0.511) after 1 h, 211 ± 122 vs. 198 ± 109 mg cumulative tramadol (P=0.610) after 4 h, 244 ± 150 vs. 231 ± 134 mg cumulative tramadol (P= 0.793) after 8 h, 250 ± 156 vs. 247 ± 153 mg cumulative tramadol (P=0.972) after 12 h, and 294 ± 190 vs. 284 ± 178 mg cumulative tramadol (P=0.791) after 24 h, postsurgery (Figure 2). Tramadol plus ramosetron was not significantly inferior to tramadol alone in alleviating the postoperative pain, since the upper and lower limits of the 95% CI of the 24h cumulative tramadol difference were 83.34 and -63.93 and the lower limit of the 95% CI was above the predetermined lower noninferiority margin (-85.20) for the 24h cumulative tramadol difference. Furthermore, there were no significant group differences in pain scores (at rest and during movement) during the study period (Figure 3). The nausea scores (0/1/2/3) 1 h after surgery were 89.8/8.16/2.04/0% and 94.12/3.92/1.96/0% in the R and N groups, respectively (P=0.437). The nausea scores (0/1/2/3) 24 h after surgery were 55.10/14.29/22.45/8.16% and 45.10/13.72/35.29/5.88% in the R and N groups, respectively (P=0.343). No group differences were observed in nausea scores (Table 1). The total fentanyl dose was slightly lower in the R group (2150 mg) compared to N group (1950 mg) in PACU, but this difference was not significant (P=0.313). A single rescue dose of Trolac 30 mg was given to three patients in R group and two patients in N group in ward. The total metoclopramide dose was slightly lower in the R group (210 mg) compared to N group (310 mg) in PACU, but this difference was not significant (P=0.437).

Furthermore, patient satisfaction with postoperative pain management did not differ between the two groups. No patient complained of headache, dizziness, or skin flushing, adverse effects known to occur with 5-HT_3_ receptor antagonists.

## 4. Discussion

This randomized, controlled trial showed that simultaneous use of ramosetron did not affect tramadol's analgesic action during postoperative pain management. In a previous trial, Kim et al. [9] reported no antagonistic effects between ramosetron and tramadol, similar to our results.

Satisfactory postoperative analgesia is very essential because it results in faster recovery of pulmonary function, early ambulation, and shorter hospital stays [11]. PCA allows patients to manage their pain well; continuous infusion of analgesics with an initial loading dose is very useful to achieve patient satisfaction [12]. The effect of IV PCA tramadol has been demonstrated to be similar to that of PCA morphine following major surgery [13]. Tramadol is associated with less euphoria and addiction, because it exerts a markedly weaker *μ*-agonist effect compared with morphine [14]. Houmes et al. [15] also reported less respiratory depression with tramadol, although the incidence of nausea and vomiting was higher. At present, 5-HT_3_ antagonists are the most widely used antiemetics for preventing PONV. Adding a 5-HT_3_ antagonist to tramadol IV PCA regimen would reduce nausea and vomiting. However, negative effects of 5-HT_3_ antagonists on the analgesic efficacy of tramadol have been reported [6–8]. For this reason, drug interaction must be considered. The mono o-desmethyl metabolite (M1) of tramadol has its analgesic effects. M1 metabolites are formed largely by CYP2D6 activity, and serotonin receptor antagonists are also metabolized by CYP2D6 isoenzyme [16, 17]. Therefore, simultaneous administration of these drugs causes competition for CYP2D6 and affects the pharmacokinetics of ondansetron and tramadol. However, an interaction between tramadol and ramosetron could be less affected by pharmacokinetics since it had been reported that ramosetron does not cause clinically important CYP-mediated drug interactions in vivo and ramosetron undergoes metabolism by CYP1A2 [18]. In various clinical trials, ramosetron has been shown to have a more potent and longer antiemetic effect compared to ondansetron [19, 20]. Roh et al. [21] demonstrated that ramosetron is superior to palonosetron, the most-recent 5-HT_3_ antagonist, for preventing PONV with intravenous PCA, despite palonosetron having greater receptor affinity and a longer duration of action. Moreover, Kim et al. [9] showed that a single dose of ramosetron had no antagonist effect against tramadol.

The dose of ramosetron used in the present study was selected based on a previous study [22, 23]. We coadministered 0.6 mg of ramosetron with intravenous-tramadol IV PCA to prevent PONV. Few studies have examined the efficacy of ramosetron combined with IV PCA for preventing PONV. Choi et al. [22] reported that adding ramosetron to PCA effectively reduced the incidence of PONV during the first 48 h after surgery. Kim et al. [23] reported that a single dose of ramosetron (0.3 mg) followed by ramosetron (0.6 mg) mixed with PCA significantly decreased PONV compared with a single dose of ramosetron (0.3 mg). Ogata et al. [24] reported that the 5-HT_3_ receptor occupancy of ramosetron diminished to 50–60% 24 h after a single injection of ramosetron, and continuous infusion of ramosetron after a bolus dose would maintain a higher 5-HT_3_ receptor occupancy level. In the present study, we used no loading dose of ramosetron before starting PCA, because we wanted to investigate the analgesic effect of tramadol with continuous coadministration of ramosetron. If we had administrated ramosetron before a loading dose of tramadol, a significant group difference in PONV incidence may have arisen.

This study had several limitations. First, we failed to reduce the nausea score and total antiemetic dose in group R. This might be explained by the fact that an emetogenic effect of tramadol involving the 5-HT_3_ receptor is expected to be most pronounced during a loading dose of tramadol. In clinical settings, patients generally tend to discontinue PCA demands if they notice a distinct correlation between PCA doses and emesis [25]. Furthermore, the power analysis was based on cumulative consumption of tramadol. Thus, the sample size was insufficient: a larger number of patients are required to assess the antiemetic efficacy of ramosetron when coadministered with tramadol. Second, we did not know the optimal dose of ramosetron for combined use with PCA. Further studies are needed to evaluate the optimal administration dose of ramosetron with intravenous PCA. Third, we used a single-blinded study design, in which investigators unblinded to the treatment received by each group assessed the pain and nausea scores, rescue antiemetic doses, and patient satisfaction. Lastly, we did not give a loading dose of ramosetron before starting the PCA, so it is unclear whether ramosetron has reached a sufficient concentration to cause drug interaction with tramadol.

In conclusion, continuous administration of ramosetron did not decrease the analgesic action of tramadol. Therefore, we concluded that ramosetron will not affect dose of tramadol.

## Figures and Tables

**Table 1:** Patients' characteristics and anesthesia data.

		Ramosetron (n=49)	Control (n=51)	P value
		N (%) or median (min-max)		
Age (years)		47 (23-60)	46 (24-64)	0.893 ^W^
Weight (Kg)		55 (45-70)	57 (46-101)	0.324 ^W^
Duration of surgery (min)		95 (30-220)	105 (30-380)	0.541 ^W^
Duration of anesthesia (min)		130 (60-285)	145 (65-420)	0.638 ^W^
Amount of infused remifentanil		496.5 (206-1334)	527 (161-2088)	0.526 ^W^
Amount of infused propofol		945 (190-2800)	1160 (481-3515)	0.169 ^W^
PONV scores 1h postoperatively	0	44 (89.8)	48 (94.1)	0.713 ^F^
	1	4 (8.2)	2 (3.9)	
	2	1 (2.0)	1 (2.0)	
PONV scores 24h postoperatively	0	27 (55.1)	23 (45.1)	0.542 ^F^
	1	7 (14.3)	7 (13.7)	
	2	11 (22.4)	18 (35.3)	
	3	4 (8.2)	3 (5.9)	
ASA	Status I	40 (81.6)	32 (62.7)	0.036 ^C^
	Status II	9 (18.4)	19 (37.3)	
Satisfaction	dissatisfied	1 (2.0)	2 (4.0)	0.881 ^F^
(missing =1)	neutral	13 (26.5)	12 (24.0)	
	satisfied	24 (49.0)	27 (54.0)	
	very satisfied	11 (22.5)	9 (18.0)	

C: Chi square test, F: Fisher's exact test, W: Wilcoxon rank sum test, PONV: postoperative nausea and vomiting, satisfaction for the overall analgesic technique.

## Data Availability

The data used to support the findings of this study are included within the article.
